# Synthesis of soluble oligsiloxane-end-capped hyperbranched polyazomethine and their application to CO_2_/N_2_ separation membranes

**DOI:** 10.1080/15685551.2018.1472720

**Published:** 2018-05-23

**Authors:** Liang Xu, Tianyang Lei, Boyu Jing, Yu Zang, Fengjuan Miao, Toshiki Aoki, Masahiro Teraguchi, Takashi Kaneko

**Affiliations:** a College of Chemistry and Chemical engineering, Qiqihar University, Qiqihar, China; b College of Materials Science and Engineering, Qiqihar University, Qiqihar, China; c College of Communications and Electronics Engineering, Qiqihar University, Qiqihar, China; d Faculty of Engineering, Niigata University, Nishi-ku, Japan

**Keywords:** Polyazomethine, hyperbranched, oligosiloxane, blend membrane, CO_2_/N_2_ separation

## Abstract

Three soluble hyperbranched polyazomethines containing oligosiloxane end group **HBP-PAZ-SiO_n_** were successfully synthesized. **HBP-PAZ-SiO_n_s** were used as modifiers of ethyl cellulose (EC) and polysulfone (PS) membranes. Blend membranes, **HBP-PAZ-SiO_n_**/EC and **HBP-PAZ-SiO_n_**/PS were prepared by blending the THF solution of **HBP-PAZ-SiO_n_** with ethanol solution of EC and dichloromethane solution of PS, respectively. Surprisingly, the permeabilities for CO_2_ of the blend membranes were more than 15–16 times higher than those of pure EC and PS membranes without any drop of pemselectivity to N_2_. This unusual improvement has been achieved by both enhancement of diffusivity for carbon dioxide and nitrogen by the oligosiloxane groups and enhancement of affinity of the amino groups with carbon dioxide at the end groups of **HBP-PAZ-SiO_n_**.

## 1. Introduction

Many gas permselective membranes have been reported in the past three decades as energy saving separation process because they can solve recent environmental problems [1–12]. Among them, membranes permeating carbon dioxide selectively are very important in view of solving the global warming problem. For example, they can eventually remove CO_2_ from flue gas [3,13–17].

In general, requirements for polymer materials as a practical gas permselective membrane are the following three: 1) a high gas permeability coefficient for a Gas A (*P*_A_ such as *P*_CO2_), 2) a high gas permselectivity of Gas A to Gas B (*P*_A_/*P*_B_ such as *P*_CO2_/*P*_N2_), and 3) good membrane-forming ability giving an ability to yield a thin membrane. However, trade-off relationships between the permeability and permselectivity have been often observed [18]. In addition, polymers which may have high permeability and pemselectivity tend to have low membrane forming ability. To overcome these problems, more precise design of chemical structures of polymers used for permselective membranes is needed. Polyazomethines (**PAZ**) are known for their excellent thermal stability, good mechanical strength, environmental resistance, and optoelectronic property [19–23]. **PAZ**s are usually synthesized from diamine and dialdehyde and therefore they have amine end groups. Since amine groups have strong interaction with CO_2_, they are useful for CO_2_ separation [16]. However, due to the aromatic conjugated structures, the solubility of aromatic **PAZ** is very low. Hyperbranched polymers (HBP) have been reported, and they have high solubility compared with the corresponding linear ones. [24–34] Since insoluble polymers are not suitable for permselective membranes materials, we selected HBP of **PAZ** (= **HBP-PAZ**).

In this paper, in order to enhance solubility of **HBP-PAZ**, we introduced oligosiloxane chains with a different length by reaction with an oligosiloxane end capping reagent to the end of **HBP-PAZ** to give **HBP-PAZ-SiO_n_**. Their performances as CO_2_ permeation membrane materials were estimated by using blend membranes of **HBP-PAZ-SiO_n_** with substrate polymers, ethyl cellulose (EC) and polysulfone (PS).

## 2. Experimental

### 2.1. Materials

All the solvents used for monomer synthesis and polymerization were distilled as usual. Melamine and isophthalaldehyde were purchased from Aladdin Industrial Corporation. The silicon containing reagents purchased from TCI chemical Co., Inc., were used as received.

### 2.2. Measurements

#### 2.2.1. Measurements of carbon dioxide and nitrogen permeability

Carbon dioxide and nitrogen permeability coefﬁcients (*P*_CO2_ and *P*_N2_: cm^3^(STP)·cm·cm^−2^·s^−1^·cmHg^−1^) and the carbon dioxide separation factor (*P*_CO2_/*P*_N2_) were measured by a gas chromatographic method by using YANACO GTR-11 MH according to our previous report. [30,32,35,36]

The mixture of carbon dioxide and nitrogen (50/50(v/v)) was used for the feed gas. The *P*_CO2_ and *P*_N2_ were calculated by the following equation:

$$P=\frac{Q\times l}{A\times \Delta p\times t}$$

where Q, *l*, A, Δp, and t are the amount of the permeated gas, the thickness of the membrane, the permeation area of the membrane, the pressure difference across the membrane and the permeation time, respectively. Disc-type membranes were used. The **A** and ***l*** of the membranes were 1.77 cm^2^ and around 120–310 μm, respectively. The **Δ** p was 1 atm and the measurement temperature was 25°C.

The schematic view of the experimental setup is shown in Figure S1. A polymer film was placed in the membrane cell and exposed to vacuum to remove the gases from the polymer and membrane cell for 10 min by pull ‘VAC’, and then, the mixed CO_2_/N_2_ test gas was feed to membrane cell through ‘In’. After the retention time and wait for steady state, the permeate gas in the ‘permeate storage tube’ was measured by gas chromatography through the time lag. The residue gas in ‘Membrane cell’ was released through ‘Retentate’. The amount of the permeated gas (Q) was calculated from the peaks of gas chromatogram.

#### 2.2.2. Other measurements

^1^H NMR (600MHz) spectra were recorded on an AVANCE III spectrometer. The average molecular weights (*M*_n_ and *M*_w_) were evaluated by gel permeation chromatography (GPC) by using Polymer Laboratories (Varoam) liquid chromatography instruments (with MIXED-E, MIXED-B, MIXED-A, MZ-Gel SDplus columns, THF eluent, polystyrene calibration). The infrared spectra were recorded on Spotlight 400.

### 2.3. Synthesis of soluble oligosiloxane-end-capped hyperbranched polyazomethines HBP-PAZ-SIO_n_

**Scheme 1.**
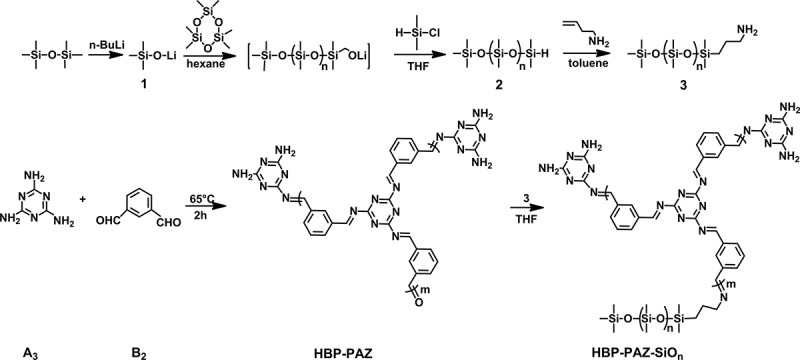
Synthesis of soluble oligosiloxane end capped hyperbranched polyazomethine **HBP-PAZ-SiO_n_** (n = 9,18 and 39).

Soluble oligosiloxane-end-capped polyazomethines **HBP-PAZ-SiO_n_** were synthesized according to Scheme 1. All the following reaction procedures were conducted under dry nitrogen.

#### 2.3.1. Synthesis of oligosiloxane end capping reagent precursor 2

n-Butyllithium (35.3 mL, 70.6 mmol, 2.0 N in hexane) was added dropwise to the tetrahydrofuran (THF) (50.0mL) solution of hexamethyldisiloxane (11.5mL, 70.6mmol) at 0℃. After refluxing for 24h, THF was removed by evaporation and the crude product **1** was purified by vacuum drying.

The mixture of hexamethylcyclotrisiloxane (D3) (n = 9: 7.25g, 37.5mmol; n = 18: 14.5g, 65.0mmol; n = 39: 28.9g, 130mmol) and cyclohexane (50.0mL) was stirred for 30min at room temperature. Then, compound **1** (1.20g, 10.0mL) was add to the mixture, and after 1h stirring at room temperature, THF(25.0mL) was add to the mixture followed by stirring at room temperature for 24h. Finally, the dimethylchlorosilane (5.01mL, 5.00mmol) was injected into the mixture and stirred for another 3h at room temperature. The mixture was filtered, THF was removed by evaporation. The crude product was purified by vacuum drying to give **2** as a yellowish transparent liquid.

**n **= 9: Yield: 65.3% (5.52 g). ^1^H NMR (CDCl_3_, ppm): *δ* = 4.70 (m, 1H, (CH_3_)_2_SiH), 0.05–0.09 (br, 69H, (CH_3_)_2_SiO).

**n **= 18: Yield: 70.7% (11.1 g). ^1^H NMR (CDCl_3_, ppm): *δ* = 4.70 (m, 1H, (CH_3_)_2_SiH), 0.05–0.09 (br, 136H, (CH_3_)_2_SiO).

**n **= 39: Yield: 49.2% (9.23 g). ^1^H NMR (CDCl_3_, ppm): *δ* = 4.70 (m, 1H, (CH_3_)_2_SiH), 0.05–0.09 (br, 273H, (CH_3_)_2_SiO).

#### 2.3.2. Synthesis of oligosiloxane end capping reagent(SIO_n_) 3

The resulting compound **2** (1.71mmol) was added to the toluene (8.10mL) solution of allylamine (759μL, 10.1mmol) and 3-divinyl-1,1,3,3,3,3-tetramethyldisiloxane platinum (0) complexes (453μL, 1.01mmol). The mixture was stirred at 45℃ for 24 h, and then, the mixture was filtered, solvent was removed by evaporation. The crude product was purified by vacuum drying to give **3** as a brown transparent liquid.

**n **= 9: Yield: 79.8% (1.42 g). ^1^H NMR (CDCl_3_, ppm): *δ* = 2.66 (t, 2H, CH_2_NH_2_), 1.45 (m, 2H, CH_2_CH_2_NH_2_), 0.53 (m, 2H, SiCH_2_CH_2_NH_2_), 0.05–0.09 (br, 75H, (CH_3_)_2_SiO).

**n **= 18: Yield: 88.0% (2.32 g). ^1^H NMR (CDCl_3_, ppm): *δ* = 2.66 (t, 2H, CH_2_NH_2_), 1.45 (m, 2H, CH_2_CH_2_NH_2_), 0.53 (m, 2H, SiCH_2_CH_2_NH_2_), 0.05–0.09 (br, 136H, (CH_3_)_2_SiO).

**n **= 39: Yield: 84.3% (4.53 g). ^1^H NMR (CDCl_3_, ppm): *δ* = 2.66 (t, 2H, CH_2_NH_2_), 1.45 (m, 2H, CH_2_CH_2_NH_2_), 0.53 (m, 2H, SiCH_2_CH_2_NH_2_), 0.05–0.09 (br, 265H, (CH_3_)_2_SiO).

#### 2.3.3. Synthesis of HBP-PAZ

A typical procedure for synthesis of **HBP-PAZ** was as follows: A solution of melamine (A_3_) (305mg, 2.43mmol) and isophthalaldehyde (B_2_) (500mg, 3.73 mmol) (A3/B2 = 0.65) in 1,3-dimethyl-2-imidazolidinone was stirred for 2h at 65℃. Then, the mixture was poured into a 100mL beaker containing 50.0mL ethyl acetate. After precipitation, the liquid was filtered. The crud solid product was purified by vacuum drying to give a white solid.

Other polymerizations of melamine and isophthalaldehyde were carried out similarly. The results are shown in Table 1.

**Table 1.** Synthesis and characterization of **HBP-PAZ.**

			Solubility (%)		
No.	/[B_2_] ^a^	Yield (%)^b^	THF	DMF	DMSO
1	0.65	58.1	19.5	18.6	20.9
2	0.60	63.3	18.9	17.6	18.4
3	0.55	68.4	13.8	11.8	20.5
4	0.50	56.6	13.4	7.80	13.2

^a^ The feed ratio of melamine (A_3_) with isophthalaldehyde (B_2_)

^b^ Insoluble part in ethyl acetate

^c^Soluble part in THF, by GPC correlating polystyrene standard (eluent: THF).

**HBP-PAZ**: IR(KBr): 3469&3133cm^−1^ (NH_2_), 1671cm^−1^ (C = O), 1543cm^−1^ (C = N), 1438cm^−1^(C-N).

#### 2.3.4. Synthesis of HBP-PAZ-SIO_n_

A typical procedure for synthesis of **HBP-PAZ-SiO_9_** was as follows: To a mixture of **HBP-PAZ** (5.00mg) and THF(1.20mL), oligosiloxane end capping reagent **3** (n = 9) (175mg, 0.200mmol) was added and refluxed for 48h. The mixture was filtered, solvent was removed by evaporation. The crude product was purified by vacuum drying to give **HBP-PAZ-SiO_9_** as a brown viscous liquid.

Other end capping reactions of **HBP-PAZ** with **3** were carried out similarly. The yields of **HBP-PAZ-SiO_n_** (n = 9, 18 and 39) were 94.5%, 94.2% and 93%, respectively.

**HBP-PAZ-SiO_n_**: IR(KBr): 3469&3133cm^−1^ (NH_2_), 1671cm^−1^(C = O), 1543cm^−1^ (C = N), 1438cm^−1^(C-N), 1263cm^−1^(Si-C), 1091cm^−1^(Si-O), 788cm^−1^ (Si-C).

### 2.4. Preparation of HBP-PAZ-SIO_n_/EC and HBP-PAZ-SIO_n_/PS blend membranes

**HBP-PAZ-SiO_n_/**EC and **HBP-PAZ-SiO_n_/**PS blend membranes, were fabricated as follows: a solution of **HBP-PAZ-SiO_n_** in THF (1.5 mg/mL) and a solution of the substrate EC in ethanol (30 mg/mL) (for PS, in CH_2_Cl_2_) were blended together, and then the resulting blend solution was cast on a Teflon sheet. After evaporating the solvent for 24h at room temperature, the membrane was detached from the sheet and dried in vacuo for 24 h. And then the carbon dioxide and nitrogen permeability were measured by a gas chromatographic method by using YANACO GTR-11 MH [35,36]. The active permeation area was 1.77cm^2^ and the thickness of the membranes were 120-310μm.

## 3. Results and discussion

### 3.1. Synthesis of oligosiloxane end-capping reagents

Three kinds of oligosiloxane end-capping reagents **SiO_n_** (n = 9, 18 and 39) were synthesized by living polymerization in the different feed ratio of hexamethylcyclotrisiloxane/lithium trimethylsilanolate with yield of 52.1, 34.7 and 34.4%, respectively. The **n** of **SiO_n_** were 9, 18 and 39 which were confirmed by the integral ratio of OSi((CH_3_)_2_) to the terminal SiH in the ^1^H NMR spectra of **2**. The observed values were consistent with the theoretical calculation values.

### 3.2. Synthesis of hyperbranched polyazomethine

**Figure 1.**
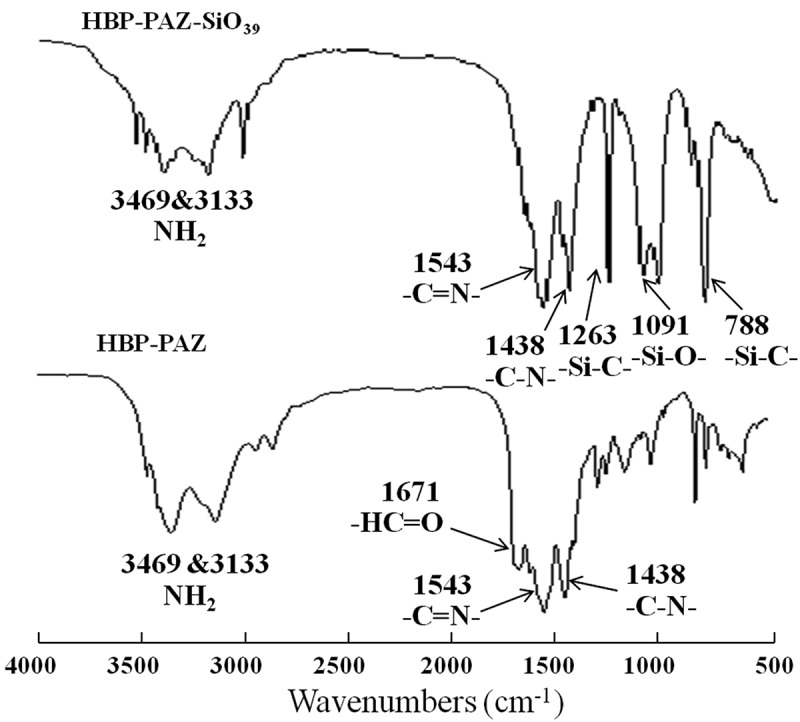
IR spectra of **HBP-PAZ** and **HBP-PAZ-SiO_39._**

Hyperbranched polyazomethines (**HPB-PAZ**) were successfully synthesized by condensation polymerization of melamine (A_3_) and isophthalaldehyde (B_2_) with yields higher than 56.6%. The chemical structures were confirmed by FT-IR spectra (Figure 1) because they had insoluble part. The -C = N- stretching vibration band around 1543cm^−1^, the NH_2_ and – HC = O stretching vibration band of end groups around 3469-3133cm^−1^ and 1671cm^−1^ were observed. It indicates the condensation polymerization between amine and aldehyde was achieved to form **HPB-PAZ**. Many amine and aldehyde end groups remain in **HPB-PAZ**.

By changing the A_3_/B_2_ feed ratio from 0.50 to 0.65, the solubility and molecular weight (THF soluble part) were changed, the results are shown in Table 1. When the A_3_/B_2_ feed ratio reaches to 0.65, the **HPB-PAZ** shows the highest solubility of 19.5% in THF and 20.9% in DMSO. The *M*_n_ and *M*_w_ values of the soluble part were also the highest among the four **HPB-PAZ**s (*M*_n_ = 3,500, *M*_w_ = 3,600, DP≈15).

### 3.3. Synthesis of soluble oligosiloxane-end-capped hyperbranched polyazomethine HBP-PAZ-SIO_n_

**Figure 2.**
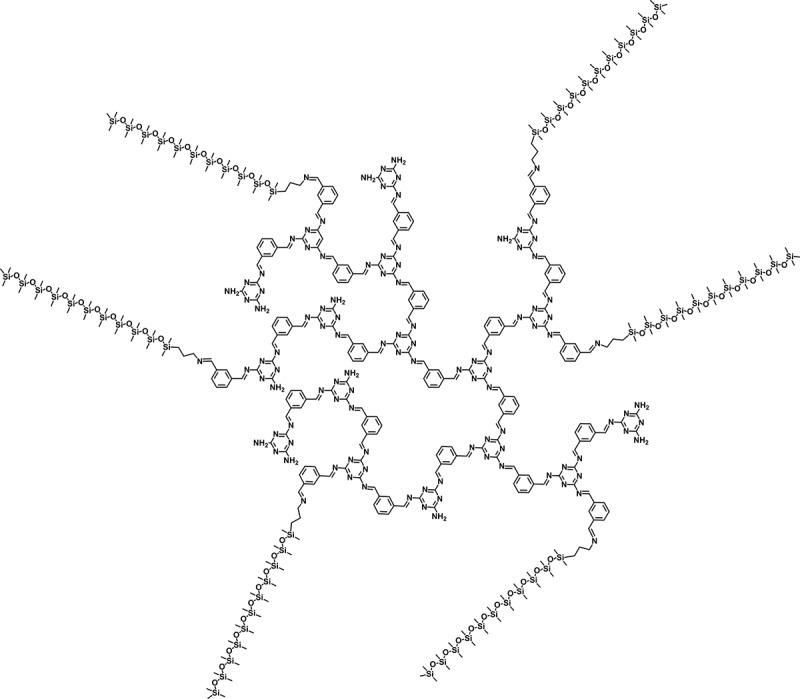
An example of the structure of **HBP-PAZ-SiO_9_** (DP = 15).

Soluble oligosiloxane-end-capped hyperbranched polyazomethines (**HBP-PAZ-SiO_n_**) were synthesized by reaction of the aldehyde end groups of **HBP-PAZ** with the amine group of the oligosiloxane end capping reagent **SiO_n_** (n = 9, 18, 39). The yields of the resulting **HBP-PAZ-SiO_n_** were higher than 94%. From the FT-IR spectra of **HBP-PAZ-SiO_39_** (Figure 1), the new Si-C and Si-O stretching vibration bands around 1263 and 1091cm^−1^ are found and the C = O stretching vibration band of the aldehyde end group of **HBP-PAZ** decreased. It indicates that the end capping reaction has been achieved. Judging from these data, an example of chemical structure is shown in Figure 2.

### 3.4. The CO_2_/N_2_ separation of HBP-PAZ-SIO_n_/EC and HBP-PAZ-SIO_n_/PS blend membranes

A 5wt% of soluble oligosiloxane-end-capped hyperbranched polyazomethine (**HBP-PAZ-SiO_n_**) was blended with EC and PS to give a self-standing membrane. The CO_2_/N_2_ permeation experiments were carried out and the results are shown in Table 2. By adding **HBP-PAZ-SiO_9_**, the permeability (*P*_CO2_) of EC and PS were enhanced and reached more than 9 times higher values without any drops of the pemselectivity (*P*_CO2_/*P*_N2_) (Table 2, Nos. 2 and 6). In addition, with increasing the length of oligosiloxane (n) of **HBP-PAZ-SiO_n_** from 9 to 39, the *P*_CO2_ increased and reached values about 15–16 times higher than those for EC and PS substrates (Table 2, Nos. 4 and 8).

**Table 2.** Carbon dioxide permeation behavior of **HBP-PAZ-SiO_n_**/EC and **HBP-PAZ-SiO_n_**/PS blend membranes.

No.	Membrane ^a^	*P*_CO2_^b^ (Barrer)	*P*_N2_^b^ (Barrer)	*P*_CO2_/*P*_N2_
1	EC	44	2.2	20
2	**HBP-PAZ-SiO_9_**/EC	400	20	20
3	**HBP-PAZ-SiO_18_**/EC	480	24	20
4	**HBP-PAZ-SiO_39_**/EC	640	33	19
5	PS	5.6	0.25	22
6	**HBP-PAZ-SiO_9_**/PS	51	2.2	22
7	**HBP-PAZ-SiO_18_**/PS	67	3.1	22
8	**HBP-PAZ-SiO_39_**/PS	88	4.1	22

^a^ EC: ethyl cellulouse; PS: polysulfone.

^b^ Barrer = 10^−10^cm^3^ (STP)·cm·cm^−2^·s^−1^·cmHg^−1^.

The extremely high enhancements of *P*_CO2_ were caused by the enhancements of diffusivity by the introduction of the **SiO_n_** chains. Although these enhancements of *P*_CO2_ were usually reported, simultaneous large decreases in *P*_CO2_^/^ *P*_N2_ were usually observed. In this study, surprisingly no decreases were observed. This may be caused by the interaction of amino groups in **HBP-PAZ-SiO_n_** with CO_2_, a week acid. The interaction avoided decreasing the selectivity.

## 4. Conclusions

Three soluble oligosiloxane-end-capped hyperbranched polyazomethines **HBP-PAZ-SiO_n_** (n = 9, 18 and 39) were synthesized by reaction of aldehyde end group of **HBP-PAZ** with the amine group of oligosiloxane end capping reagent **SiO_n_** (n = 9, 18, 39). The permeability of EC and PS substrate membranes were enhanced more than 15 times by using the **HBP-PAZ-SiO_39_** as modifiers without any drop on pemselectivity. In the three **HBP-PAZ-SiO_n_, HBP-PAZ-SiO_39_** showed the highest performance due to the longer oligosiloxane chain end. This unusual improvement has been achieved by both enhancement of diffusivity for carbon dioxide and nitrogen by the oligosiloxane groups and enhancement of affinity of the amino groups with carbon dioxide at the end groups of **HBP-PAZ-SiO_n_**.

## References

[1] Lau C, Li P, Li F, Chung T, Paul D Reverse-selective polymeric membranes for gas separations. Progr Polym Sci. 2013;38:740–766.

[2] Yampolskii Y. Polymeric gas separation membranes. Macromolecules. 1999;45:3298–3311.

[3] Sanders D, Smith Z, Guo R, Robeson L, McGrath J, Paul D, Freeman B Energy-efficient polymeric gas separation membranes for a sustainable future: a review. Polymer. 2013;54:4729–4761.

[4] Ghosal K, Freeman B Gas separation using polymer membranes: an overview. Polym Adv Technol. 1994;5:673–697.

[5] Freeman B, Pinnau I Separation of gases using solubility-selective polymers. Trends Polym Sci. 1997;5:167–173.

[6] Liaw D, Wang K, Huang Y-C, Lee K, Lai J, Ha C Advanced polyimide materials: syntheses, physical properties and applications. Prog Polym Sci. 2012;37:907–974.

[7] McKeown N, Budd P Polymers of intrinsic microporosity. ISRN Mater Sci. 2012;2012:150–151.

[8] Zhang J, Kang H, Martin J, Zhang S, Thomas S, Merkel T, Jin J The enhancement of chain rigidity and gas transport performance of polymers of intrinsic microporosity via intramolecular locking of the spiro-carbon. Chem Commun. 2016;52:6553–6556. 10.1039/c6cc02308h 27103254

[9] Rose I, Carta M, Malpass-Evans R, Ferrari M, Bernardo P, Clarizia G, Jansen J, McKeown N Highly permeable benzotriptycene-based polymer of intrinsic microporosity. ACS Macro Lett. 2015;4:912–915. 10.1021/acsmacrolett.5b00439 35596456

[10] Yampolskii Y A current position of polyacetylenes among other highly permeable membrane materials. Polym Rev. 2017;57:200–212.

[11] Aoki T Macromolecular design of permselective membranes. Progr Polym Sci. 1999;24:951–993.

[12] Zang Y, Aoki T, Teraguchi M, Ferrari M, Bernardo P, Clarizia G, Jansen J, McKeown N Synthesis and oxygen permeation of novel polymers of phenylacetylenes having two hydroxyl groups via different lengths of spacers. Polymer. 2015;56:199–206.

[13] Adewole J, Ahmad A, Ismail S, Leo C Current challenges in membrane separation of CO_2_ from natural gas: A review, Int J Green Gas Con. 2013;17:46–65.

[14] Lin H, Freeman B Materials selection guidelines for membranes that remove CO_2_ from gas mixtures. J Mol Struct. 2005;739:57–74.

[15] Liu J, Hou X, Park HB, Lin H High-performance polymers for membrane CO_2_/N_2_ separation. Chem Eur J. 2016;22:15980–15990. 27539399 10.1002/chem.201603002

[16] Taniguchi I, Duan S, Kazama S, Fujioka Y Facile fabrication of a novel high performance CO_2_ separation membrane: immobilization of poly(amidoamine) dendrimers in poly(ethylene glycol) networks. J Membr Sci. 2008;322:277–280.

[17] Vinoba M, Bhagiyalakshmi M, Alqaheem Y, Alomair A, Pérez A, Rana M Recent progress of fillers in mixed matrix membranes for CO_2_ separation: A review. Sep Purif Technol. 2017;188:431–450.

[18] Robeson L The upper bound revisited. J Membr Sci. 2008;320:390–400.

[19] Iwan A An overview of LC polyazomethines with aliphatic-aromatic moieties: thermal, optical, electrical and photovoltaic properties. Renew Sust Energ Rev. 2015;52:65–79.

[20] Iwan A, Sek D Processible polyazomethines and polyketanils: from aerospace to light emitting diodes and other advanced applications. Prog Polym Sci. 2008;33:289–345.

[21] Iwan A, Sek D Polymers with triphenylamine units: photonic and electro-active materials. Prog Polym Sci. 2011;36:1277–1325.

[22] Jeevadason A, Murugavel K, Neelakantan M Review on Schiff bases and their metal complexes as organic photovoltaic materials. Renew Sust Energ Rev. 2014;36:220–227.

[23] Hussein M, Abdel-Rahman M, Asiri AM, Asiri A, Alamry K, Aly K Review on: liquid crystalline polyazomethines polymers. Basics, syntheses and characterization. Des Monomers Polym. 2012;15:431–463.

[24] Voit B, Lederer A Hyperbranched and highly branched polymer architectures synthetic strategies and major characterization aspects. Chem Rev. 2009;109:5924–5973. 19785454 10.1021/cr900068q

[25] Gao C, Yan D Hyperbranched polymers: from synthesis to applications. Prog Polym Sci. 2004;29:183–275.

[26] Kwak G, Masuda T Synthesis, characterization and optical properties of a novel Si-containing σ-π-conjugated hyperbranched polymer. Macromol Rapid Commun. 2002;23:68–72.

[27] Gradwell S, Kepler L Preparation of novel photoluminescent oligocarbosilanes by hydrosilylation. Macromolecules. 2002;35:2871–2872.

[28] Kwak G, Masuda T Synthesis and thermal properties of regio- and stereoregular poly(silylene-1,4-phenylenevinylene)s. Macromol Rapid Commun. 2001;22:1233–1236.

[29] Lach C, Frey H Enhancing the degree of branching of hyperbranched polymers by postsynthetic modification. Macromolecules. 1998;31:2381–2383.

[30] Wang J, Li J, Aoki T, Kaneko T, Teraguchi M, Shi Z, Jia H Subnanoporous highly oxygen permselective membranes from poly(conjugated hyperbranched macromonomer)s synthesized by one-pot simultaneous two-mode homopolymerization of 1,3-bis(silyl)phenylacetylene using a single Rh catalytic system: control of their structures and permselectivities. Macromolecules. 2017;50:7121–7136.

[31] Li J, Wang J, Zang Y, Aoki T, Kaneko T, Teraguchi M Enhanced gas permselectivity of copoly(hyperbranched macromonomer) synthesized by one-pot simultaneous copolymerization of dimethylsilyl-containing phenylacetylenes. Chem Lett. 2012;41:1462–1464.

[32] Kaneko T, Yamamoto K, Asano M, Teraguchi M, Aoki T Synthesis of poly(phenylacetylene)-based polydendrons consisting of a phenyleneethynylene repeating unit, and oxygen/nitrogen permeation behavior of their membranes. J Membr Sci. 2006;278:365–372.

[33] Aoki T, Kaneko T, Teraguchi M Synthesis of functional π-conjugated polymers from aromatic acetylenes. Polymer. 2006;47:4867–4892.

[34] Kaneko T, Asano M, Yamamoto K, Aoki T Polymerization of phenylacetylene based monodendrons and structure of the corresponding polydendrons. Polym J. 2001;33:879–890.

[35] Kawakami Y, Aoki T, Hisada H, Yamamura Y, Yamashita Y Poly(p-disiloxane substituted styrene)s as materials for oxygen permeable membranes. Polym Commun. 1985;26:133–136.

[36] Aoki T, Toyoshima Y, Oikawa E Synthesis of poly[p-(1H,1H,2H,2H-perfluoroalkyloxyoligosiloxanyl)styrene]s and oxygen permselectivity of their membranes. Polym J. 1994;26:1142–1153.

